# What Proportion of Female Sex Workers Practise anal Intercourse and How Frequently? A Systematic Review and Meta‑analysis

**DOI:** 10.1007/s10461-019-02477-w

**Published:** 2020-03

**Authors:** Branwen Nia Owen, Rebecca F. Baggaley, Jocelyn Elmes, Amy Harvey, Zara Shubber, Ailsa R. Butler, Romain Silhol, Peter Anton, Barbara Shacklett, Ariane van der Straten, Marie‑Claude Boily

**Affiliations:** 1 Department of Infectious Disease Epidemiology, Imperial College London, Norfolk Place, St Mary’s Campus, Praed Street, London W2 1NY, UK; 2 Department of Global Health and Development, London School of Hygiene and Tropical Medicine, London, UK; 3 Department of Medicine, David Geffen School of Medicine, UCLA, Los Angeles, USA; 4 Department of Medical Microbiology and Immunology, University of California, Davis, USA; 5 Women’s Global Health Imperative Program, RTI International, San Francisco, USA; 6 Department of Medicine, Center for AIDS Prevention Studies, UCSF, San Francisco, USA

**Keywords:** Anal intercourse, Female sex workers, Sexual behavior, HIV

## Abstract

HIV is more efficiently acquired during receptive anal intercourse (AI) compared to vaginal intercourse (VI) and may contribute substantially to female sex workers’ (FSW) high HIV burden. We aim to determine how common and frequent AI is among FSW globally. We searched PubMed, Embase and PsycINFO for studies reporting the proportion of FSW practising AI (prevalence) and/or the number of AI acts (frequency) worldwide from 01/1980 to 10/2018. We assessed the influence of participant and study characteristics on AI prevalence (e.g. continent, study year and interview method) through sub-group analysis. Of 15,830 identified studies, 131 were included. Nearly all (N = 128) reported AI prevalence and few frequency (N = 13), over various recall periods. Most studies used face-to-face interviews (N = 111). Pooled prevalences varied little by recall period (lifetime: 15.7% 95%CI 12.2–19.3%, N= 30, I^2^ = 99%; past month: 16.2% 95%CI 10.8–21.6%, N= 18, I2 = 99%). The pooled proportion of FSW reporting < 100% condom use tended to be non-significantly higher during AI compared to during VI (e.g. any unprotected VI: 19.1% 95%CI 1.7–36.4, N = 5 and any unprotected AI: 46.4% 95%CI 9.1–83.6, N = 5 in the past week). Across all study participants, between 2.4 and 15.9% (N = 6) of all intercourse acts (AI and VI) were anal. Neither AI prevalence nor frequency varied substantially by any participant or study characteristics. Although varied, AI among FSW is generally common, inconsistently protected with condoms and practiced sufficiently frequently to contribute substantially to HIV acquisition in this risk group. Interventions to address barriers to condom use are needed.

## Introduction

HIV is very effectively transmitted during anal intercourse unprotected by condoms (UAI), with a meta-analysis finding that women may have an 18-fold greater HIV acquisition risk during UAI compared to vaginal intercourse unprotected by condoms (UVI) [1]. Thus, even a small proportion of intercourse acts being AI may therefore substantially contribute to HIV transmission [2, 3]. However, the role of anal intercourse (AI) within heterosexual epidemics has not been sufficiently examined and is frequently overlooked [4]. For example, recent reviews on HIV risk behaviour among female sex workers (FSW) in China [5] and among young people in Africa [6] examined multiple measures of sexual risk-taking but neither included AI practice. Likewise, public health messaging to FSW on HIV transmission seems to routinely neglect AI practice. For example, none of the studies included in two systematic reviews on HIV prevention interventions among African FSW reported whether or not messaging on safe AI was included in the interventions [7, 8]. This omission may contribute to the lack of awareness of transmission risk during AI among FSW [3, 9] and subsequently to condoms being used less consistently during AI compared to VI (vaginal intercourse) [3, 10].

The practice of AI among FSW has been reported in many articles. However, the extent to which AI is practised by FSW and how often it is practised by age, region and over time has yet to be comprehensively described. It is particularly pertinent to examine these patterns among FSW, compared to other population groups, as FSW experience a far greater burden of HIV and STI infection than women in the general population [11]. This review will be useful to improve our understanding of AI practices, inform prevention messages and identify knowledge gaps. Parameter estimates derived from this review can be used in mathematical models to explore the contribution of AI to the HIV epidemic and assess the influence of AI on the predicted effectiveness of prevention interventions.

In order to estimate the contribution of AI to HIV and STI incidence among FSW and transmission to their sexual partners, it is first necessary to accurately described AI practice in this group. To estimate this contribution, we need data on the proportion of FSW who practise AI and at what frequency, with which types of partner AI is practised and whether condoms are used [4]. The equivalent information for VI is required for a complete understanding of an individual’s potential HIV risk through heterosexual sex. Our review aims to systematically review and summarise published estimates on the proportion of FSW reporting AI and the number of AI acts, and to examine the sources of variation in AI practice.

## Methods

The systematic review was undertaken following PRISMA guidelines for reviews of observational studies [12].

### Search Strategy

PubMed, Embase and PsycINFO were searched for English-language articles published 1st January 1980 to 31st October 2018 reporting on sexual behaviour among FSW (see Supplement A for full search terms). The screening of identified records was conducted by only one reviewer; with BNO conducting the search from 1990 onwards alone and JE from 1980 to 1989. We did not include the term ‘anal’ in our search to avoid rejecting studies that, while eligible, did not refer to AI in the title or abstract. We discarded titles that were obviously irrelevant, then screened abstracts and retrieved full-text articles if any sexual behaviour among FSW (defined as exchanging sexual services for payment, either cash or in-kind) was reported. Bibliographies of included articles were scanned for further relevant articles. Studies were included in the review if they fulfilled the following criteria:

Published, peer-reviewed articles on cross-sectional studies, cohort studies or randomised control trials (RCTs) that reported data on FSW from which it was possible to extract or calculate the proportion practising AI and/ or the number of AI and UAI acts over any recall period.

Although grey literature can be useful, its inclusion can introduce difficulties in ensuring that the search is systematic and that the studies included are methodologically sound. We therefore chose to restrict our review to capture the highest quality peer-reviewed evidence available using an easily replicable search strategy.

### Data Extraction

We defined a priori the variables to be extracted. We used a standard procedure to extract data to a spreadsheet. Each publication was examined by two reviewers independently, with differences resolved by consensus. The intra-class correlation coefficient (ICC) was calculated for each outcome of interest to estimate inter-rater reliability. Our outcomes of interest were (1) AI prevalence (the proportion of participants reporting practising AI), (2) monthly frequency of AI and VI, (3) fraction of all intercourse acts and all unprotected intercourse acts which are AI and UAI (details of how these were derived are in Supplement B and C). We extracted participant and study characteristics, including measures of study quality (listed in Table 1, with the addition of alcohol and drug use and sexual and physical violence victimisation). Baseline data only were extracted from longitudinal studies and unadjusted estimates were extracted from studies using respondent-driven sampling. We contacted authors of included studies when key variables of interest were not reported.

### Data Synthesis and Statistical Methods

#### Prevalence Data

We produced forest plots of individual study estimates for the most common recall periods. We calculated overall pooled estimates and 95% confidence intervals (95%CI) for AI prevalence across each available recall period. As our review includes diverse populations of FSW, we anticipated substantial heterogeneity in AI prevalence estimates across studies. We therefore pooled results using random-eff models and conducted extensive sub-group analysis to explore sources of heterogeneity [13–15]. Sub-group analysis on the effect of participant characteristics and study characteristics on pooled AI prevalence estimates were conducted for recall periods with over 10 estimates. Continuous variables were dichotomised at the median. To compare condom use during AI and VI we calculated the proportion reporting any UAI among those reporting AI, as well as the equivalent for VI. We plotted these individual study estimates and produced pooled estimates by recall period (for recall periods with > 3 estimates). Where studies reported condom use as ‘always’, ‘sometimes’ or ‘never’, rather than over a specific recall period, we define answers other than ‘always’ as practising UAI or UVI and refer to this recall period as general condom use. All models were fitted using maximum-likelihood random-effects models [16, 17] with the procedure ‘Metafor’ [18] in R version 3.20.1 [19]. Heterogeneity across study estimates was investigated using Cochran’s Q test and its *p* value [20] as well as I^2^ estimates [21].

#### Frequency Data

To enable comparison across studies which reported number of AI acts by different recall periods, we standardised frequency estimates to number of acts per month. Where possible, we derived the proportion of all intercourse acts that were AI or UAI. When the mean number of AI acts was reported only among the sub-samples who practise AI, we also derived the mean among the whole sample, when AI prevalence was also reported. As very few studies reported measures of variance of intercourse act data, we were unable to conduct statistical synthesis of frequency data; thus, we limited our analysis to graphically exploring the effects of participant and study characteristics on the proportion of intercourse acts that were anal.

### Dealing with Bias

Our sub-group analyses included exploring the effect of different measures of methodological quality; interview method, study design, recruitment method and response rate. We also examined through sub-group analysis the section in the article where AI was first mentioned (title, abstract or main text), which we used to explore the possible effect of publication bias as authors may be more likely to include or highlight AI data when the practice is more common.

## Results

### Search Results

Figure S1 summarises the study selection procedure and search results. Of the 13,658 unique articles initially identified 131 were included. Most articles were identified from the database searches, and two were identified through reference scanning. Additional information was obtained from 23 of the 35 authors contacted. Inter-rater reliability for the outcomes of interest was high, with ICC ranging from 0.85 for AI frequency data to 0.96 for AI prevalence data.

### Study and Participant Characteristics

Details of each included study are presented in Table SI and participant and study characteristics are summarised in Table 1. AI prevalence was reported over various recall periods by 128 studies (including five studies reporting UAI prevalence only [22–25] with five comparing AI prevalence over two or more recall periods [3, 9, 29–31]. The most common AI prevalence recall periods were lifetime (N = 30) and 1 month (N = 18). A very large number of studies failed to state the recall period at all (N = 52); these included 35 studies which reported whether FSW provided AI as part of their service. AI frequency data (either number of AI acts and/or the proportion of intercourse acts which were AI) was provided by only 13 studies.

Sample sizes ranged from 12 to 9667 for a total sample size of 74,426 across all studies (Table SI). Nearly half of the studies specified partner type, with 15 reporting AI practice separately for non-paying partners and paying clients. Most studies were conducted in Asia (N= 53), followed by Africa (N = 34) and Europe (N = 23), with few conducted in the Americas (N = 14 in North, N= 10 in South America, respectively). Median age across studies was 28 years and median survey year 2003. The vast majority of studies either did not report location of work (N = 53) or reported on samples with a mixture of indoor and outdoor sex workers (N = 38).

We were unable to include the use of alcohol (reported by 23 studies, or drug use (reported by 20 studies) or physical and sexual violence (reported by 12 and 11 studies, respectively) in our analysis, because they were too rarely reported and when reported, used a wide range of recall periods.

### Study Quality and Potential Bias

More studies reported on FSW who worked only indoors (N = 33), than outdoors (N = 12) (Table 1). Most studies used face-to-face interviews (FTFI) (N = 111), were cross-sectional in design (N = 116) and employed convenience sampling (N = 96). Three studies compared the reporting of AI practice by interview method [23, 26, 27]. Most failed to report the response rate (N = 110). More studies first mentioned AI in the main text (N = 88), than abstract (N = 32) or title (N = 11) (Table 1).

### Meta‑analysis of AI Prevalence

**Fig. 1:**
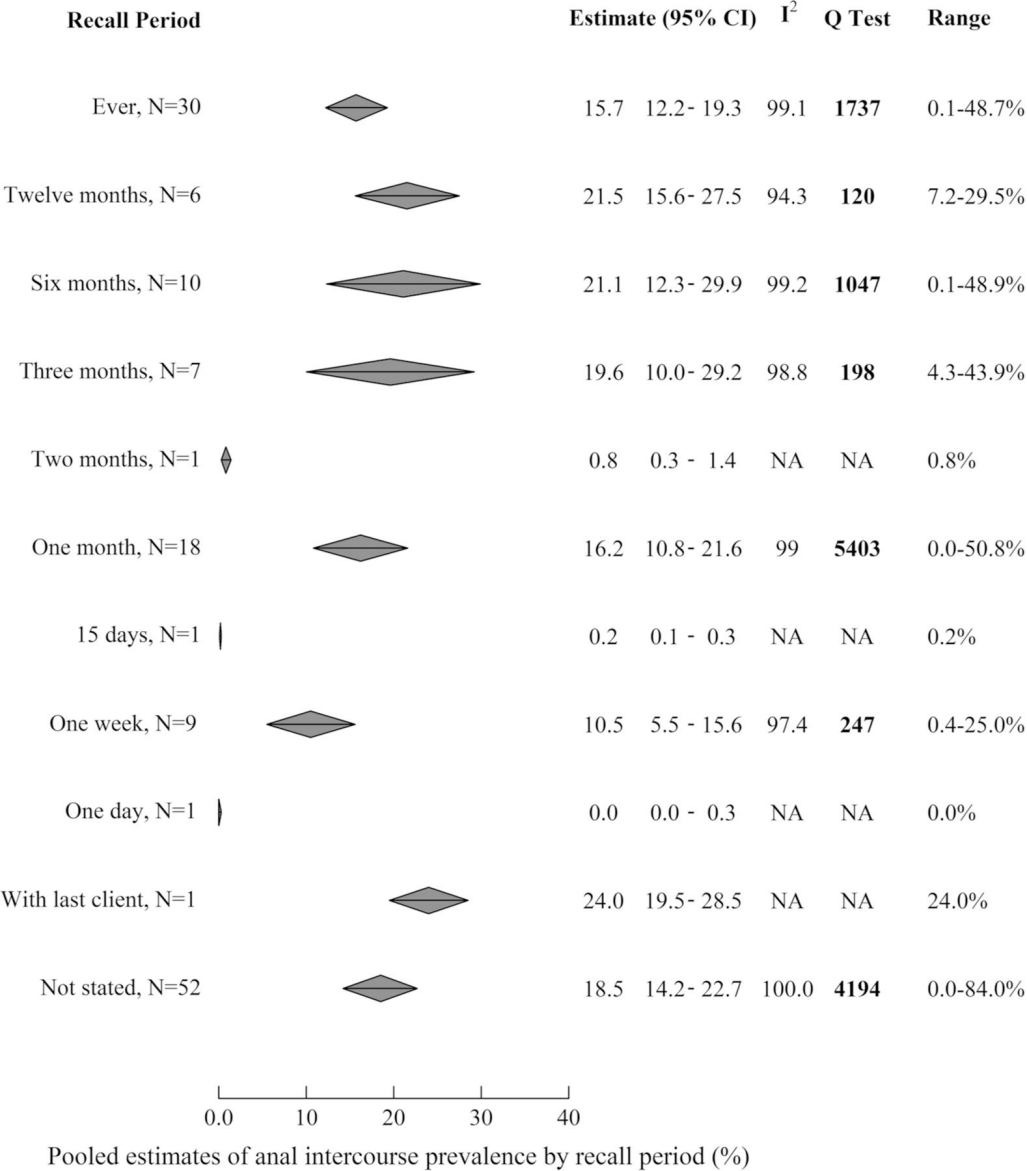
Pooled estimates of the prevalence of anal intercourse over each recall period reported. *AI* anal intercourse, *NA* not applicable, *95% CI* 95% confidence interval. The top of each diamond represents the pooled estimate, while furthest points represent 95% CI. I^2^ and Q Test are both measures of heterogeneity, with higher values in both indicating greater heterogeneity. I^2^ ranges from 0–100%. The results of the Q Test are displayed in bold when the *p*-value is < 0.05, which indicates that the level of heterogeneity found is statistically significant

Figure 1 displays pooled estimates of AI prevalence for all recall periods and Fig. S2a–c displays individual study estimates for the three most common recall periods (lifetime and past month), respectively. Reported AI prevalence varied substantially between studies, ranging from 0.0 to 84.0% across recall periods (Table S1). Estimates stratified by recall period remained very heterogeneous (I^2^ > 90% and all Q tests showing statistically significant heterogeneity). Pooled AI prevalence did not vary substantially by length of recall period apart from 2 months, 15 days and 1 day recall periods, which all only had one study each (Fig. 1). Aside from these, pooled estimates varied between 10.5% (95%CI 5.5–15.6%, N = 8) in the past week and 21.5% (95%CI 15.6–27.5%, N = 6) in the past year, and the pooled estimate for reporting ever having practiced AI was 15.7% (95%CI12.2–19.3).

### Sub‑group Analysis of AI Prevalence

Table 2 shows pooled estimates from sub-group analyses of AI prevalence by participant and study characteristics for recall periods with sufficient numbers of study estimates (ever and past 1 month).

#### Participant Characteristics

Pooled estimates of lifetime AI practice tended to be higher among older FSW [28+ years= 20.7% (95%CI 14.5–26.9%, N= 13) vs. < 28 years = 11.9% (95%CI 7.9–15.9%, N= 14)], in studies conducted after 2002 (2003 onwards = 19.2% (95%CI 15.4–24.8%, N = 18) vs pre-2003 = 12.9% (95%CI 5.3–19.2%, N = 13). The same patterns were seen for AI practice in the past month, but as with lifetime prevalence, differences between sub-groups were not significant. Pooled estimates did not vary by partner type, continent, average number of clients or location of work.

#### Study Quality and Bias

Pooled estimates of lifetime and past month prevalence for cross-sectional studies were lower compared to estimates from RCT and cohort studies, respectively. However, these observations are inconclusive as there was only one RCT and one cohort study reported lifetime and past month prevalence, respectively. Pooled estimates of lifetime and 1 month AI practice was higher when the word ‘anal’ was first mentioned in the article title compared to in the abstract or main text [e.g. for lifetime, title = 23.9% (95%CI 14.0–33.8%, N = 4) versus text = 13.2% (95%CI 8.0–18.3%, N = 17)]. Pooled estimates did not vary by interview method, recruitment method or response rate.

### Comparative Condom use During AI and VI

**Fig. 2:**
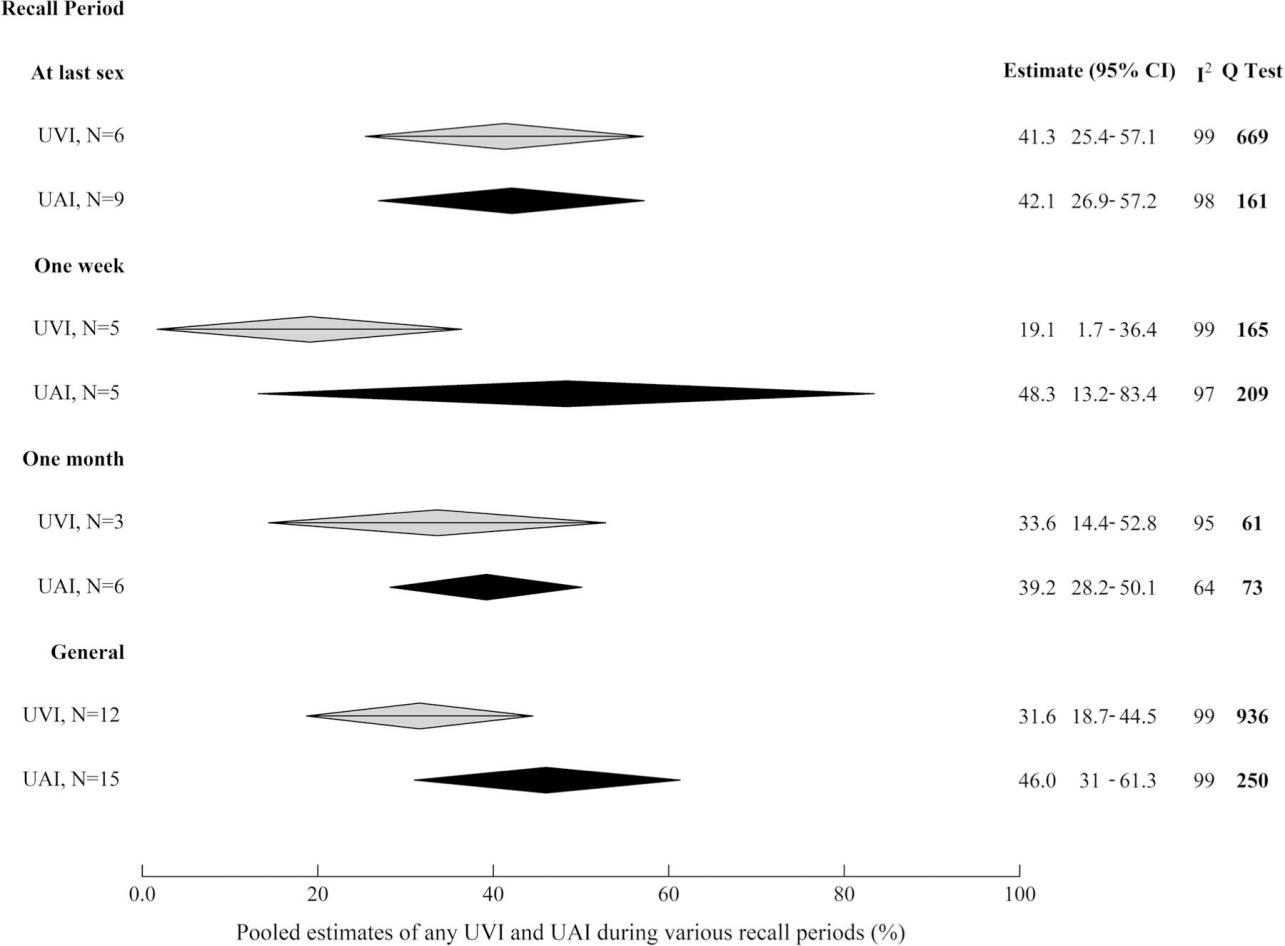
Pooled estimates of the prevalence of anal intercourse and vaginal intercourse unprotected by condoms, by recall period. Pooled estimates of the proportion of those who report any AI unprotected by condoms among those reporting any AI over the most commonly reported recall periods, and the equivalent pooled estimates for UVI. *UAI* anal intercourse unprotected by condoms, *UVI* vaginal intercourse unprotected by condoms, *95% CI* 95% confidence interval, *general* report that condom use is anything other than ‘always’ using condoms

Pooled estimates of the prevalence of UAI among those reporting AI were higher than UVI among those reporting VI in four of the five recall periods over which it was reported (Fig. 2) [e.g. general UAI = 46.0% (95%CI 30.8–61.3), UVI = 31.6% (95%CI 18.7–44.5)], although 95%CIs overlapped substantially (individual study estimates are plotted in Fig. S3a–d).

### Frequency of AI Compared to VI

**Fig. 3:**
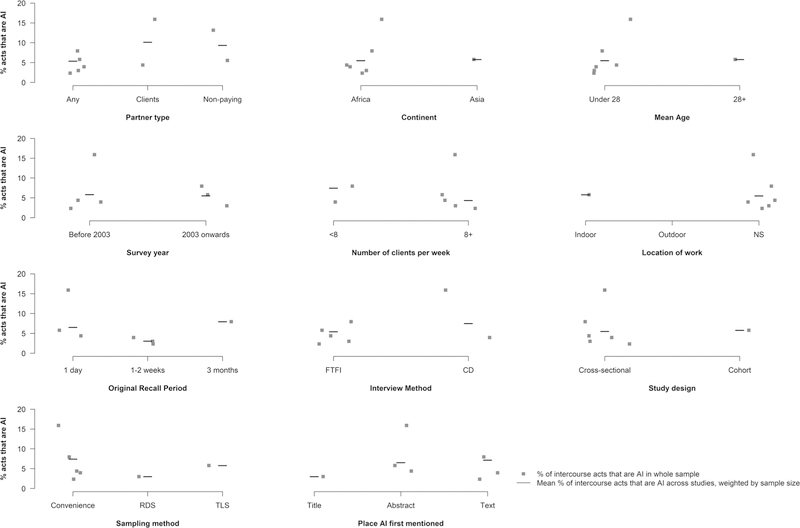
Proportion of intercourse acts that are anal by selected study and participant characteristics Scatter plots of the proportion of intercourse acts that are anal among the whole sample (i.e. including those reporting no AI) participant characteristics and study characteristics. *ACASI* audio computer assisted self-interview, *CD* coital diary, *CRS* cluster-randomised sampling, *FTFI* face-to-face interview, *Mix* data only available for men and women combined, *NS* not stated, *RCT* randomised controlled trial, *RDS* respondent-driven sampling, *SAQ* self-administered questionnaire, *SRS* simple randomised sampling, *TLS* Time-location sampling

Of the 13 studies which provided data on the number of AI acts, we were able to extract or derive eight estimates among the subset of FSW who report practising AI [3, 9, 10, 28–32] and eight over the whole sample [3, 10, 26, 32–36], which includes FSWs not practising AI (Table 3). AI frequency estimates vary substantially across studies. Across the studies providing data among the subset of FSWs reporting AI, the number of AI and UAI acts per month ranged from 1.8 to 27.8 (N = 8) and from 0.2 to 6.2 (N = 3), respectively. Among studies reporting mean frequency across the whole study sample, the total number of AI and UAI acts ranged from 1.1 to 16.9 (N = 8) and 1.0 to 1.7 (N = 3). The percentage of all intercourse acts that were anal ranged from 2.4 to 15.9% in the six studies that reported it across the whole sample [3, 26, 33–36]. In the sole study which reported it among the subset practising AI [3], 17.0% of intercourse acts were anal. The proportion of intercourse acts that were anal did not vary substantially by any participant or study characteristics (Fig. 3).

## Discussion

This extensive review adds to the current literature and understanding of AI practices among FSW. We found that reported AI practice is generally common among FSW worldwide, with a pooled estimate of 15.7% (95%CI 12.2–19.3) ever having practised AI. There was substantial heterogeneity across study estimates that largely was not explained by any of the measured participant and study characteristics. AI tended to be more often unprotected by condoms compared to VI, although this was not statistically significantly different. Although scarce, the available data on AI frequency suggests that AI is practised frequently, with 2.4–15.9% of all intercourse acts being anal among all FSW study participant samples.

Similar to previous review findings regarding heterosexual AI practice among young people and South Africans [37, 38], we found a non-statistically significant indication that AI prevalence may have increased over time. In qualitative research Indian and East African FSW have described AI practice during sex work as becoming more common over time due to increased client demand [9, 39–41]. Pooled AI prevalence varied little across recall periods and in the four studies which reported AI practice over multiple recall periods AI prevalence changed little as recall periods lengthened [3, 28, 42, 43]. These findings suggest that those who initiate AI continue to practise it.

The strengths of our study include conducting a wide search and identifying a large number of eligible studies, resulting in a large sample size. Our review was greatly strengthened by using wide search terms, for example, omitting the word ‘anal’, ensured that we captured eligible studies which first mentioned AI in the main text, rather than the title or abstract. Given that AI prevalence tended to be lower the later in the article that AI was first mentioned, our search strategy limited the impact of publication bias, thus increasing the accuracy of our results. Deriving estimates for AI practice where possible also helped reduce publication bias. We conducted a detailed sub-group analysis to identify potential sources of heterogeneity in AI practice based on characteristics measured in the study, including measures of study quality.

Our review has a number of limitations. We did not include articles in languages other than English, or grey literature, which may have resulted in omission of potentially eligible articles. Our language restriction resulted in the exclusion of 42 potentially eligible full-text articles. Eleven percent full-text articles examined were found to be eligible, and if the same proportion of identified non-English full-text articles were eligible, this would have resulted in the inclusion of an additional four or five studies to our review. However, the language restriction is unlikely to have influenced results substantially given the large number of articles included (N = 131). We searched for grey literature in our similar review of heterosexual AI among South Africans [37] and found none eligible.

Our review was mainly limited by the quality of reporting on AI practice. Of the 131 included studies, 52 failed to report the recall period of AI prevalence. Only a third of studies reporting AI prevalence also provided data on condom use during AI as well as VI. Only 10% (13 of 131 studies) of included studies reported any type of AI frequency data, and a single study provided the number of each type of intercourse act necessary to fully describe AI frequency (number of anal and vaginal acts over the same recall period, both condom protected and not) [36]. Only two studies [3, 26] provided standard deviation or 95%CI for intercourse act data, which prevented us from pooling the few data available.

AI is a highly stigmatised behaviour in many societies and thus its reporting is likely subject to social desirability bias and is likely more accurately reported using more confidential interview methods [37, 38]. As the majority of studies in this review used FTFI, the least confidential interviewing method, our pooled estimates of AI prevalence and estimates of AI frequency likely underestimate its practice among FSW. Our sub-group analysis found that AI prevalence was not higher in the small number of studies which used more confidential methods compared to those that used FTFI. However, the two included studies which compared AI prevalence by interview method both found non-significantly higher prevalence using more confidential methods compared to FTFI [23, 27]. One study in this review compared AI frequency by interview method, finding more than five times as many anal intercourse acts were reported by FSW in South Africa when using coital diaries compared to daily FTFI [26].

### Recommendations for Future Reporting of AI Practice

It is clear from this review and others [37, 38] that data collection on AI practice requires improvement, especially given how effectively HIV is transmitted during AI and how commonly it is practiced. Previous research suggests that survey items must be carefully piloted in order to minimise misunderstanding and that one effective approach may be the use of pictograms to unambiguously clarify what is meant by AI [44]. Using confidential interview methods would help reduce social-desirability bias.

We need data that paints a complete picture of AI practice and which allows the proportion of all intercourse acts that are anal to be estimated. Accurately estimating this proportion is key to estimating the extent to which AI impacts on HIV epidemics among FSW [4]. In order to minimise bias when estimating the fraction of intercourse acts that are AI, the same recall period should be used to collect data on AI and VI practice. We recommend that the following questions be included in all surveys on sexual behaviour among FSW:

- Have you had AI in the past 12 months?
- How many VI acts have you had in the past week with (a) clients and (b) non-paying partners?
- Was a condom used throughout your last VI act with (a) a client and (b) a non-paying partner
- How many AI acts have you had in the past week with (a) clients and (b) non-paying partners?
- Was a condom used throughout your last AI act with (a) a client and (b) a non-paying partner

These recall periods may not be suitable for all FSW populations. In the case of low client volume, for example, we recommend collecting data on the number of intercourse acts over the past month. Equivalent questions should also be included in surveys among general population men and women, although past month may be a more suitable recall period for intercourse act data.

### Public Health Implications

This review provides valuable information that can be used to guide policy, research and survey design internationally, as well as to inform future mathematical models of HIV epidemics among FSW and to predict the influence that AI practice may have on intervention effectiveness. Our review has found that, while varied, AI is commonly and frequently practised by FSW, and that condoms are often less consistently used during AI compared to VI. As such, AI may substantially contribute to HIV epidemics among FSW and their sexual partners. Messaging on safe AI practice is often absent from current interventions among FSW, but should be included [39, 45, 46]. As practice of AI by FSW is most often driven by client demand [9, 39, 40, 47], programmes should address the social and environmental factors which contribute to vulnerability and hinder negotiation of safe practice; as well as target clients with safe AI messages.

## Supplementary Material

S1

S2

## Figures and Tables

**Table 1:** Summary of (A) study and participant characteristics and (B) quality of included studies

	N = 129	Sources
(A) Outcomes and key study characteristics		
Outcomes reported^a^		
AI prevalence	123	[3, 9, 10, 27–34, 42, 43, 45, 47–155]
UAI prevalence only^b^	5	[22–25, 156]
AI frequency	13	[3, 9, 10, 26, 28–36]
AI prevalence recall period^a^		
Lifetime	30	[9, 28–30, 33, 42, 50, 51, 54, 57, 60, 63, 64, 69, 79, 84, 87, 100, 104, 107, 120–122, 125, 126, 128, 130, 137, 139, 141]
12 Months	6	[3, 42, 47, 90, 105, 146]
6 Months	10	[25, 75, 80, 83, 86, 99, 110, 114, 138, 148]
3 Months	7	[22, 27, 48, 58, 62, 106, 144]
2 Months	1	[113]
1 Month	18	[3, 9, 10, 23, 28, 32, 43, 45, 59, 82, 85, 94, 95, 98, 99, 134, 143, 155]
15 days	1	[65]
7 days	9	[3, 43, 68, 82, 108, 119, 123, 127, 156]
1 day	1	[67]
With last client	1	[154]
Current primary partner	3	[29, 76, 117]
Not stated	52	[24, 29, 31, 49, 52, 53, 55, 56, 61, 66, 70–74, 76–78, 81, 88, 89, 91–93, 96, 97, 101–103, 109, 111, 112, 115–118, 124, 129, 131–133, 135, 136, 140, 142, 145, 147, 149–153]
AI practice reported by partner type^a^		
With any type	63	[3, 9, 10, 25–28, 30, 32–36, 43, 47–52, 54–56, 58, 63, 64, 66, 69, 75, 77, 79, 84, 90, 92, 98, 100–108, 110, 113, 118, 119, 122, 125, 126, 128, 130, 132, 134, 136, 137, 139, 141–143, 146–148, 150, 152, 155]
Clients^c^	62	[22–24, 31, 42, 53, 57, 59–62, 65, 67, 68, 70–74, 76, 78, 80–83, 85–89, 91, 93–97, 99, 109, 111, 112, 114–117, 120, 121, 123, 124, 127, 129, 131, 133, 135, 138, 140, 144, 145, 149, 151, 153, 154, 156]
One-time or new clients	3	[3, 29, 45]
Regular clients	3	[3, 29, 45]
Primary or non-paying partner[s)	15	[3, 29, 45, 76, 82, 83, 87, 95, 99, 117, 121, 133, 144, 151, 156]
Continent^a^		
Africa	34	[3, 10, 26–29, 34–36, 45, 48–56, 89, 104, 105, 118, 119, 125, 126, 130, 136, 141, 146, 150, 153–155]
Asia	53	[9, 10, 23, 30, 33, 42, 47, 57–60, 62–65, 67–70, 72, 73, 76, 83–85, 88, 90, 92, 94, 95, 97, 98, 100, 102, 103, 109–116, 122–124, 132, 134, 135, 140, 143, 144, 147]
Europe	23	[24, 31, 32, 71, 74, 77–82, 86, 93, 108, 117, 120, 127, 129, 133, 137, 138, 145, 148]
South America	10	[61, 66, 87, 93, 101, 121, 131, 139, 142, 152]
North America	14	[22, 25, 43, 75, 91, 96, 99, 106, 107, 128, 149, 151, 156]
Mean age^a,d^		
< 28 years	71	[3, 10, 25, 26, 28, 34–36, 48–50, 53–57, 64–69, 71–73, 75, 81–83, 85–87, 89–91, 93, 94, 96, 98, 100, 103–110, 112, 113, 117–119, 122, 124–126, 130–132, 134–136, 138, 140–142, 145, 146, 150, 154]
28+ years	57	[9, 10, 22–25, 27, 29, 31–33, 42, 43, 45, 47, 48, 52, 53, 58–60, 62, 63, 73, 74, 76–80, 84, 88, 92, 93, 95, 97, 99, 101, 102, 111, 114–116, 121, 123, 127, 128, 132, 135, 137, 139, 144, 147, 149, 151, 153, 155, 156]
Not stated	6	[30, 51, 61, 70, 120, 152]
Survey year^d^		
Pre-2003	64	[10, 26, 29, 34, 35, 49, 50, 52–56, 61, 66–70, 73–82, 88, 89, 91–94, 96, 101, 107, 108, 113, 115, 117, 119, 120, 122, 124, 125, 128–135, 137–139, 145, 146, 148–152]
2003 onwards	67	[3, 9, 22–25, 27, 28, 30–33, 36, 42, 43, 45, 47, 48, 51, 57–60, 62–65, 71–73, 83–87, 90, 95, 97–100, 102–106, 109–112, 116, 118, 121, 123, 126, 127, 136, 140–144, 147, 153–156]
Workplace^a^		
Indoors	33	[33, 52, 57, 67, 69, 72, 78, 80, 85, 87, 92–94, 98, 102, 103, 105, 108, 109, 112, 113, 128, 130–132, 134–137, 140, 146, 147, 156]
Outdoors	12	[10, 49, 53, 56, 79, 93, 108, 117, 120, 127, 135, 149]
Mixed indoors and outdoors	38	[25, 30, 35, 36, 47, 50, 58–61, 63, 65, 68, 71, 73, 82, 86, 89, 91, 96, 100, 101, 110, 111, 114, 118, 121, 122, 124, 126, 132, 141–143, 150, 153–155]
Not stated	53	[3, 9, 10, 22–24, 26–29, 31, 32, 34, 42, 43, 45, 48, 51, 54, 55, 62, 64, 66, 70, 74–77, 81, 83, 84, 88, 90, 95, 97, 99, 104, 106, 107, 115, 116, 119, 123, 125, 129, 133, 138, 139, 144, 145, 148, 151, 152]
Mean number of clients per week^a,d^		
< 10	45	[9, 25, 27, 29, 35, 36, 42, 47, 48, 54, 57, 60, 65, 81, 83–85, 89, 90, 94, 96, 97, 99, 100, 104, 109, 112–116, 119, 121–124, 126, 128, 129, 140, 141, 146, 148, 151, 156]
10 +	46	[3, 10, 25, 26, 31, 33, 34, 45, 49, 50, 52, 53, 55, 56, 58, 59, 61–63, 66–69, 71, 74, 77, 78, 80, 85, 86, 92, 102, 103, 108, 127, 132–135, 137, 139, 142, 147, 149, 152, 153]
Not stated	40	[10, 22–24, 28, 30, 32, 43, 51, 53, 64, 70, 72, 73, 75, 76, 79, 87, 88, 91, 93, 95, 98, 101, 105–107, 110, 111, 117, 118, 120, 125, 129, 136, 138, 143, 144, 150, 154, 155]
(B) Study quality and potential for bias		
Interview method^a^		
ACASI	10	[22, 27, 28, 42, 83, 86, 106, 107, 144, 155]
SAQ	5	[31, 91, 112, 133, 145]
SAQ or FTFI^e^	2	[73, 81]
FTFI	111	[3, 9, 23–27, 29, 30, 32–34, 36, 45, 47–72, 74–80, 82, 84, 85, 87–90, 92–105, 108–111, 113–132, 134–143, 153, 154, 156]
Coital diary	4	[10, 26, 35, 43]
Polling box	1	[23]
Study design		
Cross-sectional	116	[3, 9, 22–24, 26–32, 34–36, 42, 43, 47, 48, 50, 51, 53–63, 65–68, 70–74, 76–86, 89–91, 93–118, 120–128, 130–137, 139–145, 153–156]
Cohort^f^	11	[25, 33, 45, 49, 64, 69, 75, 88, 119, 129, 138]
Randomised-controlled trial^f^	4	[10, 52, 87, 92]
Sampling method		
Convenience	96	[10, 22, 24–27, 31, 32, 34–36, 43, 45, 48–51, 53–56, 58, 60, 62, 65–69, 74–82, 88, 89, 91–94, 96–103, 106, 108, 110–135, 137–139, 142–145, 154–156]
Simple-randomised sampling	5	[29, 70, 87, 132, 136]
Cluster-randomised sampling	7	[9, 57, 72, 84, 85, 107, 109]
Respondent-driven sampling	19	[3, 23, 28, 42, 52, 59, 61, 64, 71, 73, 83, 86, 90, 95, 104, 105, 140, 141, 153]
Time-location sampling	4	[30, 33, 47, 63]
Response rate		
< 90%	9	[29, 62, 67, 71, 93, 98, 112, 114, 121]
90%+	12	[9, 58, 63, 92, 94, 101, 110, 111, 115, 118, 126, 144]
Not stated	110	[3, 10, 22–25, 27, 28, 31, 32, 34–36, 42, 43, 45, 47–51, 53–56, 66, 68–70, 77–84, 88–90, 94–97, 99–105, 107–109, 113, 116, 117, 119, 120, 122–125, 128–143, 145–153, 155–157]
Place in paper where AI is first mentioned		
Title	11	[3, 9, 22, 29, 45, 47, 48, 51, 53, 83, 84]
Abstract	32	[25–28, 30, 33, 52, 57–65, 67, 71–76, 85–87, 91, 98, 106, 127, 130, 154]
Text	88	[10, 23, 24, 31, 32, 34–36, 42, 43, 49, 50, 54–56, 66, 68–70, 77–82, 88–90, 92–97, 99–105, 107–126, 128, 129, 131–153, 155, 156]

*AI* anal intercourse, *UAI* unprotected anal intercourse, *ACASI* audio-computer assisted self-interview, *FTFI* face-to-face interview, *SAQ* self-administered questionnaire

a The sum is greater than the total number of included studies because several studies provided AI data in more than one category

b Studies which reported AI prevalence for unprotected AI only

c Not specified whether one-of or regular

d Numerical variables were dichotomised at the median

e Depending on participant preference/ability

f Baseline data only extracted

**Table 2:** Sub-group analysis of AI prevalence over the most common recall periods, by participant and study characteristics

Study characteristics	Ever				Past month			
	N	Pooled estimate (95% CI)		I^2^	N	Pooled estimate (95% CI)		I^2^
*Participant characteristics*								
Partner type								
Any	25	14.8%	(11.0−18.6)	99	15	15.1%	(8.8−21.6)	99
Clients	6	19.7%	(11.3−28.0)	97	6	24.0%	(13.9−34.1)	99
New clients	0	–	–	–	2	20.3%	(8.7−32.0)	90
Regular clients	0	–	–	–	2	24.8%	(10.0−39.5)	94
Non-paying partners	2	43.9%	(14.7−73.1)	97	5	16.5%	(11.4−21.6)	83
Continent								
Africa	10	15.1%	(8.8−21.4)	98	7	20.4%	(10.1−31.8)	98
Asia	13	14.5%	(10.2−18.8)	99	12	14.0%	(6.3−21.6)	99
Europe	3	8.0%	(1.9−14.0)	86	2	21.4%	(12.9−29.8)	64
South America	3	22.2%	(14.3−30.2)	82	0	–	–	–
North America	2	29.1%	(1.8−56.3)	95	2	18.4%	(10.4−26.4)	0
Mean age								
< 28 years	14	11.9%	(7.9−15.9)	98	10	15.5%	(5.4−25.6)	99
28+ years	13	20.7%	(14.5−26.9)	99	12	18.3%	(13.2−24.0)	95
Not stated	4	10.8%	(4.3−17.3)	98	1	11.4%	(7.1−15.7)	–
Survey year								
Pre-2003	13	12.9%	(5.3−19.2)	99	7	10.5%	(1.0−19.9)	99
2003 onwards	18	19.2%	(15.4−24.8)	98	16	19.4%	(13.2−26.0)	98
Workplace								
Indoors	7	21.4%	(12.2−30.5)	94	5	14.4%	(0.0−33.8)	99
Outdoors	2	5.5%	(0.0−11.7)	86	1	40.6%	(33.6−47.7)	–
Mixed	10	8.8%	(4.8−12.8)	98	4	13.3%	(11.1−16.1)	1
Not stated	12	20.0%	(15.7−24.3)	97	13	16.8%	(11.6−22.0)	96
Number of clients/week								
< 8	12	18.6%	(10.5−26.7)	99	5	13.6%	(7.1−20.0)	97
8+	9	13.5%	(10.6−16.5)	84	10	19.6%	(9.3−29.9)	99
Not stated	10	14.3%	(9.8−18.8)	97	8	15.2%	(9.0−21.5)	96
*Study quality and potential for bias*								
Interview method								
ACASI	3	19.3%	(9.8−28.7)	95	2	11.3%	(2.7−16.3)	98
SAQ	0	–	–	–	0	–	–	–
FTFI	28	15.4%	(11.6−19.1)	99	15	17.0%	(10.3−23.6)	99
SAQ/FTFI	0	–	–	–	0	–	–	–
Coital diary	0	–	–	–	5	15.4%	(2.9−27.9)	97
Polling box	0	–	–	–	1	26.0%	(20.8−31.3)	NA
Study design								
Cross-sectional	26	15.4	(11.4−19.4)	99	14	17.5%	(11.4−23.5)	99
Cohort	3	15.0	(10.3−19.8)	57	1	37.0%	(30.3−43.7)	NA
RCT	1	31.9	(23.6−40.3)	NA	1	14.1%	(11.7−16.6)	NA
Recruitment method								
Convenience	16	13.2%	(8.3−18.1)	98	16	13.9%	(7.3−21.3)	99
Simple randomised	2	36.4%	(30.2−42.5)	12	0	–	–	–
Cluster randomised	5	14.8%	(10.9−18.9)	96	3	26.9%	(7.8−46.1)	99
Respondent-driven	5	17.8%	(9.9−25.6)	96	6	17.1%	(12.5−21.7)	90
Time-location	3	13.7%	(10.2−17.2)	90	0	–	–	–
Response rate								
< 90%	2	18.9%	(8.3–29.5)	99	1	10.2%	(7.0–14.4)	NA
90+	3	12.9%	(4.1–21.8)	99	1	13.3%	(10.5–16.2)	NA
Not stated	25	15.3%	(11.6–19.1)	99	16	16.6%	(13.5–25.8)	99
AI first mentioned								
Title	4	23.9%	(14.0–33.8)	97	3	23.8%	(12.8–34.7)	95
Abstract	10	16.9%	(13.4–20.5)	95	5	20.1%	(6.0–34.2)	99
Text	17	13.2%	(8.0–18.3)	99	15	14.1%	(8.1–20.2)	99

I^2^ is a measure of heterogeneity which can lie between 0% and 100%; with higher percentages indicating greater heterogeneity

Studies provided one estimate of AI prevalence with the following exceptions: Among studies reporting lifetime AI prevalence Kinsler et al. and Hakre et al. [87, 121] provided estimates by partner type. Among studies reporting one month AI prevalence Priddy et al., Kazerooni et al., Ojeda et al. and Maheu et al. [3, 45, 95, 99] provided estimates by partner type and Hanck et al. [23]. by interview method. Multiple study estimates per study were used only when the estimates belonged to different categories e.g. if AI prevalence estimates were available with clients and non-paying partners, then both were included in the partner type sub-group analysis, otherwise only the single estimate with the highest denominator was used

*AI* anal intercourse, *ACASI* audio-computer assisted self-interview, *FTFI* face-to-face interview, *SAQ* self-administered questionnaire, *95% CI* 95% confidence interval

**Table 3:** Frequency of anal intercourse acts, standardised per month and fraction of reported vaginal and anal intercourse acts that are anal

	Country	N	Interview method	Partner type	AI prevalence (recall period)	Number of acts/month				Original recall period	% acts that are:		% acts condom protected during:	
					%	AI	VI	UAI	UVI		AI	UAI^c^	AI	VI
*Intercourse acts reported among sub-sample who report practicing AI*														
Van Damme [10]	Multiple^a^	765	Coital diary	Any	14 (1month)	8.7	NS	NS	NS	1 week	NS	NS	NS	NS
Schwandt [29]	Kenya	147	FTFI	Any	41 (ever)	3.4	NS	NS	NS	1 month	NS	NS	NS	NS
Markosyan [32]	Armenia	98	FTFI	Any	28 (1month)	7.4	NS	6.2	NS	1 month	NS	NS	83.8	NS
Bradley [30]	India	2394	FTFI	Any	10 (ever)	8.5	NS	2.6	NS	1 week	NS	NS	30.9	NS
Hladik [28]	Uganda	942	ACASI	Any	15 (1month)	3.0	NS	NS	NS	1 month	NS	NS	NS	NS
Tucker [9]	India	555	FTFI	Any	13 (1month)	1.8	NS	0.2	NS	1 month	NS	NS	11.1	NS
Marek [31]	Hungary	34	SAQ	Clients	50 (service)	27.8	NS	NS	NS	1 day	NS	NS	NS	NS
Maheu-Giroux [3]	Cote d’Ivoire	466	FTFI	Any	19 (1month)	NS	NS	NS	NS	1 week	17.0	NS	NS	NS
*Intercourse acts reported among whole sample (i.e. including also those who report no AI)*														
Van de Perre [34]	Rwanda	33	FTFI	Any	NA	1.1	43.9	NS	NS	past 5–10 sexual encounters	2.4	NS	NS	NS
Van Damme [10]^b^	South Africa	187	Coital diary	Any	41 (1month)	4.0	NS	1.0	NS	1 month	NS	NS	25.0	NS
Ramjee [26]	South Africa	52	Weekly FTFI	Any	NS	3.0^d,e^	12.6	NS	NS	1 week	19.4	NS	NS	NS
		25	Daily FTFI	Clients	NS	3.5^d,f^	75.4	NS	NS	1 day	4.4	NS	NS	NS
		25	Daily FTFI	Primary	NS	_0.9_^d^,^g^	14.7	NS	NS	1 day	5.6	NS	NS	NS
		25	Coital diary	Clients	NS	16.9^d,h^	89.3	NS	NS	1 day	15.9	NS	NS	NS
		25	Coital diary	Primary	NS	4.3	28.6	NS	NS	1 day	13.1	NS	NS	NS
Voeten [35]	Kenya	64	Coital diary	Any	NS	1.5	37.5	NS	NS	2 weeks	4.0	NS	NS	NS
Markosyan [32]	Armenia	98	FTFI	Any	28 (1month)	2.0	NS	1.7	NS	1 month	NS	NS	85.0	NS
Carney [36]	South Africa	457	FTFI	Any	NS	2.6	30.0	1.0	9.6	3 months	8.0	3.1	38.6	32.0
Bradley [33]	India	223	Telephone^b^	Any	19 (ever)	2.9	47.0	NS	NS	1 day	5.9	NS	NS	NS
Maheu-Giroux [3]	Cote d’Ivoire	466	FTFI	Any	19 (1month)	4.3^d,i^	138.6	NS	NS	1 week	3.0	NS	NS	NS

*AI* anal intercourse, *NS* not stated, *UAI* unprotected anal intercourse, *UVI* unprotected vaginal intercourse, *VI* vaginal intercourse

a South Africa, Cote d’Ivore, Benin and Thailand

b Baseline data, including AI prevalence was collected through FTFI, all sex act data was collected via subsequent daily telephone calls

c Percentage of all intercourse acts, both protected and unprotected that are UAI

d 95%CI for intercourse act data provided:

e 95%CI 0.0–7.4.

f 95%CI 0.0–11.3.

g 95%CI 0.0–3.5.

h 95%CI 0.0–32.0.

i 95%CI 4.3–8.7
