# Supplementary material for: What Proportion of Female Sex Workers Practise anal Intercourse and How Frequently? A Systematic Review and Meta-analysis
Source: AIDS Behav. 2019 Apr 5;24(3):697–713. doi: 10.1007/s10461-019-02477-w (PMC6778486; doi:10.1007/s10461-019-02477-w)
Supplement: Supplementary file 1 — Supplementary material 1 (DOCX 2046 kb) [file 10461_2019_2477_MOESM1_ESM.docx]

## SUPPLEMENTARY INFORMATION: METHODS

## SECTION A: SEARCH DETAILS

PubMed, Embase and PsycINFO were searched from January 1^st^ 1980 to October 31^st^ 2018. A mixture of keywords and subject headings (underlined below) were used. PubMed was searched using the following terms:

(FSW[All Fields] OR (("female"[MeSH Terms] OR "female"[All Fields] OR ("female"[All Fields] AND "sex"[All Fields]) OR "female sex"[All Fields]) **AND** ("workers"[All Fields])) OR ("sex workers"[MeSH Terms] OR ("sex"[All Fields] AND "workers"[All Fields]) OR "sex workers"[All Fields]) OR ("sex workers"[MeSH Terms] OR ("sex"[All Fields] AND "workers"[All Fields]) OR "sex workers"[All Fields] OR "prostitutes"[All Fields]) OR (transactional[All Fields] AND ("sex"[MeSH Terms] OR "sex"[All Fields] OR "sex traders"[All Fields]))) AND (("behaviour"[All Fields] OR "behavior"[MeSH Terms] OR "behavior"[All Fields]) OR ("risk"[MeSH Terms] OR "risk"[All Fields])) **AND** (("sexual behavior"[MeSH Terms] OR ("sexual"[All Fields] AND "behavior"[All Fields]) OR "sexual behavior"[All Fields] OR "sexual"[All Fields]) OR ("sex"[MeSH Terms] OR "sex"[All Fields]) OR ("sexual behavior"[MeSH Terms] OR ("sexual"[All Fields] AND "behavior"[All Fields]) OR "sexual behavior"[All Fields] OR "sexually"[All Fields])) **AND** (("surveys and questionnaires"[MeSH Terms] OR ("surveys"[All Fields] AND "questionnaires"[All Fields]) OR "surveys and questionnaires"[All Fields] OR "survey"[All Fields]) OR ("clinical trials as topic"[MeSH Terms] OR ("clinical"[All Fields] AND "trials"[All Fields] AND "topic"[All Fields]) OR "clinical trials as topic"[All Fields] OR "trial"[All Fields]) OR ("cohort studies"[MeSH Terms] OR ("cohort"[All Fields] AND "studies"[All Fields]) OR "cohort studies"[All Fields] OR "cohort"[All Fields]) OR ("cross-sectional studies"[MeSH Terms] OR ("cross-sectional"[All Fields] AND "studies"[All Fields]) OR "cross-sectional studies"[All Fields] OR ("cross"[All Fields] AND "sectional"[All Fields]) OR "cross sectional"[All Fields]) OR longitudinal[All Fields])

Embase and PsycINFO were searched using these terms:

(sex work* OR prostitut* OR transaction* OR commercial* OR exchang* OR sell* OR trade* OR trading OR sex industry OR survival sex OR sex for survival OR sex trader* OR sex work/ OR sex workers/) **AND**(behaviour* OR behavior* OR risk OR exp risk/ OR exp behavior/) **AND**(sexual OR sex OR sexually OR sex/ OR exp sexual behavior/) **AND** (survey OR trial OR cohort OR cross-section* OR longitudin* OR exp "surveys and questionnaires"/ OR (surveys AND questionnaires) OR "surveys and questionnaires" OR survey OR trial OR cohort OR cross-sectional OR longitudinal OR prospective OR prevalen* OR inciden* OR exp Epidemiologic Study Characteristics as Topic/ OR Control Groups/ OR cross-over studies/ OR double-blind method/ OR lost to Follow-Up/ OR matched-pair analysis/ OR random allocation/ OR sample size/ OR "sensitivity and specificity"/ OR single-blind method/ OR "Clinical Trial, Phase III"/ OR "Clinical Trial, Phase IV"/ OR "Controlled Clinical Trial "/ OR "Observational Study"/ OR "Comparative Study"/ OR "Evaluation Studies"/ OR "Multicenter Study"/ ) **AND**(female* OR women OR woman)

## SECTION B: PREVALENCE DATA

For prevalence data we extracted, or where necessary calculated, the numerator, denominator and proportion of respondents reporting AI and VI over a given recall period, as well as those reporting any AI or VI unprotected by condoms.

### B.1 AI prevalence

Proportion reporting AI,$p$, was calculated using the following equation:

$p=\frac{n}{d}$ [1]

Where $n$ (numerator) is the number of respondents reporting AI over the recall period and $d$ (denominator) is the total number of respondents to that question. If the denominator for the question was not available, the total number of study participants was used. If the numerator was not available, it was derived from $p$ and $d$ using equation [1].

### B.2 Confidence interval for AI prevalence

Confidence intervals for prevalence estimates were calculated as follows:

$$CI=p\pm1.96\cdot\sqrt{\frac{p(1-p)}{d}}$$

Where CI is 95% confidence interval, *p* is proportion reporting AI, *d* is the number of question respondents, or if not available, then the number of study participants.

### B.3 Prevalence of AI unprotected by condoms (UAI) among those reporting AI

Proportion reporting UAI, *p*, was calculated using the following equation:

$$p=\frac{n}{d}$$

Where $n$ is the number of participants reporting UAI over the recall period and $d$ is the number of participants reporting AI. The equivalent equation was used for calculating the prevalence of vaginal intercourse unprotected by condoms (UVI).

## SECTION C: FREQUENCY DATA

## C.1 Standardising number of AI acts per month

Where number of intercourse acts was reported over recall periods other than one month, we standardised by either dividing by the number of months reported or multiplying the number of weeks to produce estimates of acts per month. A month was assumed to have 4.3 weeks and 30.5 days and a working week was assumed to have five days.

### C2 Fraction of intercourse acts that are AI

When a study did not report the fraction of intercourse acts that was AI, or unprotected intercourse acts that were UAI directly, and sufficient information was provided, we derived these as the ratio of the mean number of AI or UAI and mean number of all intercourse acts or of all unprotected intercourse acts, respectively.

$$F_{A}=\frac{\bar{{ai}_{A}}}{\bar{{ai}_{A}}+\bar{{vi}_{A}}}$$

Where $F_{A}$ is the fraction of intercourse acts that are AI, $\bar{{ai}_{A}}$ is the mean number of AI acts and $\bar{{vi}_{A}}$ is the number of VI acts.

### C.3 Fraction of intercourse acts that are UAI

$$F_{UAI}=\frac{\bar{{ai}_{U}}}{\bar{{ai}_{U}}+\bar{{vi}_{U}}}$$

Where $F_{UAI}$ is the fraction of condom unprotected intercourse acts that are UAI, $\bar{{ai}_{U}}$ is the mean number of UAI acts per person, $\bar{{vi}_{U}}$ is mean number of UVI acts per person.

### C.4 Mean number of AI acts among the whole sample, when reported among the sub-sample who report practising AI

$$\bar{a_{w}}= \bar{a_{s}} . p$$

Where $\bar{a_{w}}$ is the mean number of AI acts per person for the whole sample, $\bar{a_{s}}$is the mean number of AI acts per person in the sub-sample who report practising AI and $p$ is the proportion of the whole sample who practise AI (AI prevalence). The equivalent equation is used to calculate number of vaginal intercourse acts.

**FIGURE LEGENDS**

**Fig. S1: Summary of article search and selection.**

*Non-relevance was defined as not referring to sexual behaviour among FSW.

Two of the included studies were identified through reference scanning, with the remainder identified through the database searches.

**Fig. S2: Forest plots of the prevalence of anal intercourse over the most common recall periods**

Study estimates of AI prevalence among FSW over a) lifetime, b) one month. Estimates are ordered by survey year and grouped by partner type. I^2^ lies between 0 and 100%; 0% indicates no observed heterogeneity and larger values show increasing heterogeneity. 95%CI=95% confidence interval, ACASI=audio computer assisted self-interview, FTFI=face-to-face interview, SAQ=self-administered questionnaire

**Fig. S3: Individual study estimates the percentage reporting any AI and VI unprotected by condoms over the most common recall periods**.

Dot plots of the percentage of FSW reporting any AI unprotected by condoms among those who report practicing AI, a) generally (i.e. reporting ´sometimes or ´never´ using condoms during AI, b) in the past month, c) in the past week and d) at last intercourse act. The equivalent available estimates for VI of also plotted. The bars joining the UVI and UAI estimates are to visually aid comparison.

Fig. S1


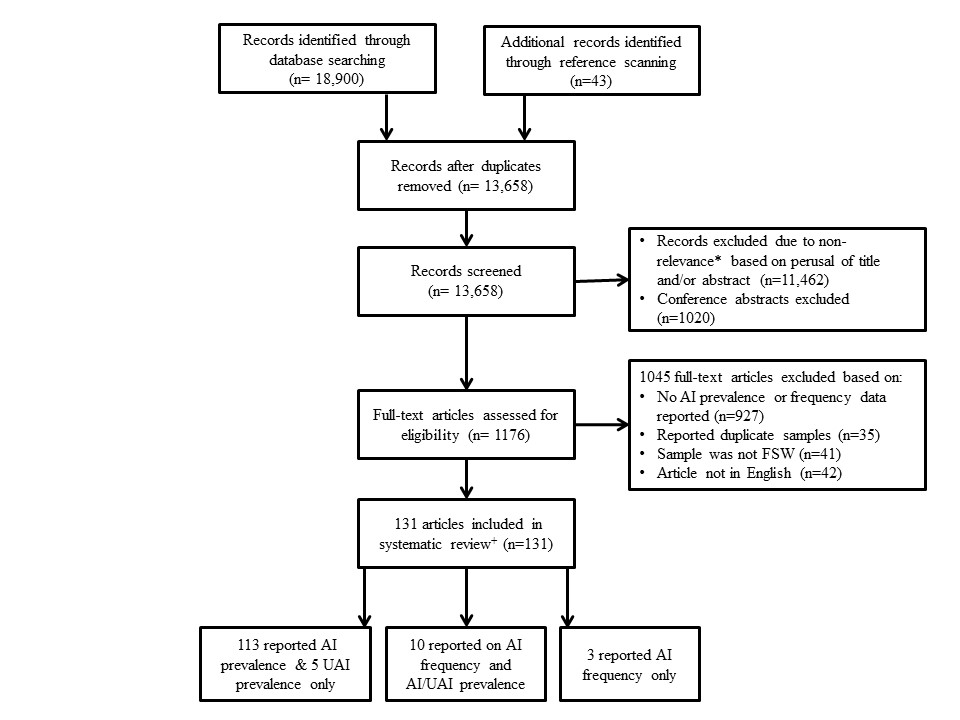


| Fig. S2a |
| --- |
| 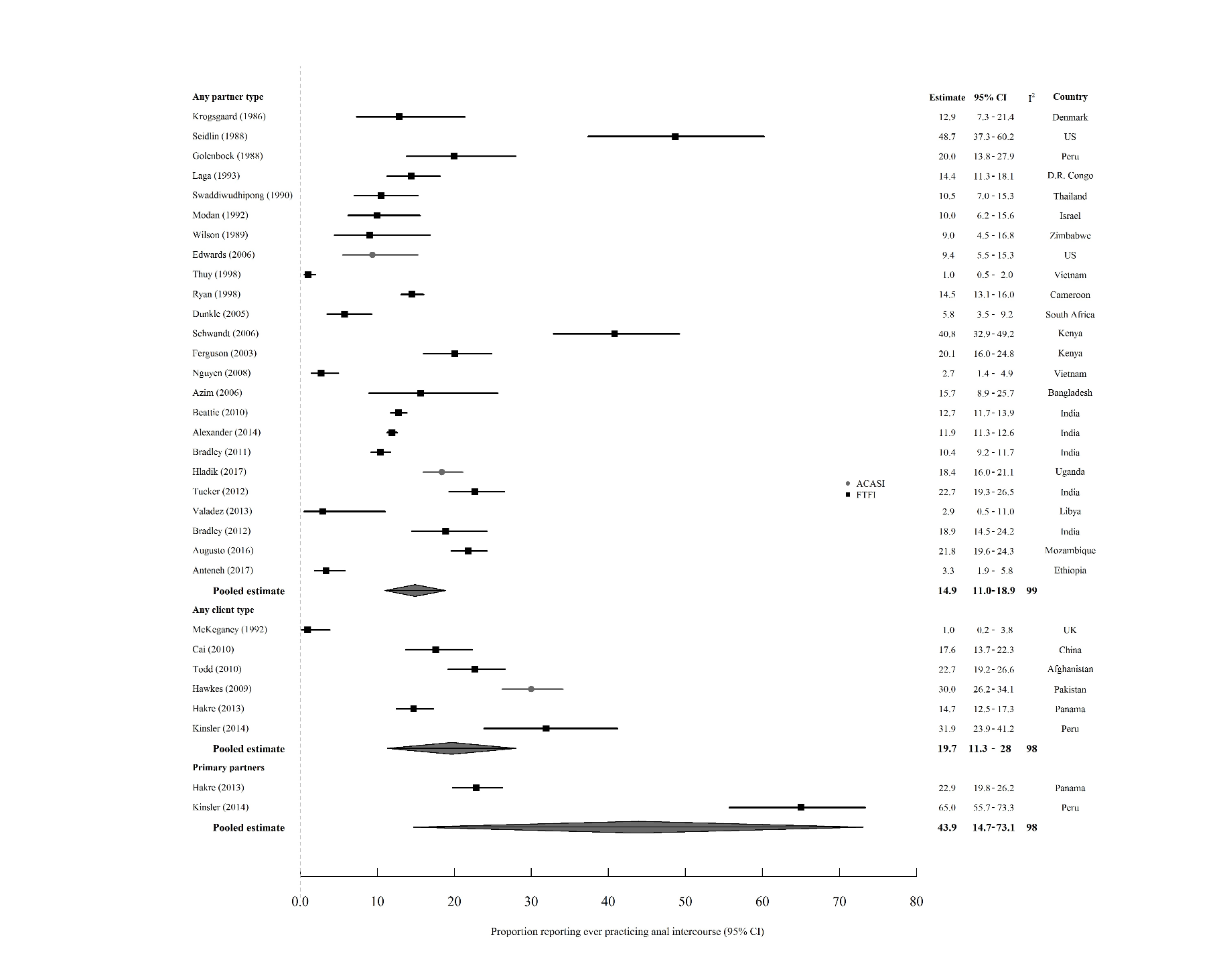 |

| Fig. S2b |
| --- |
| 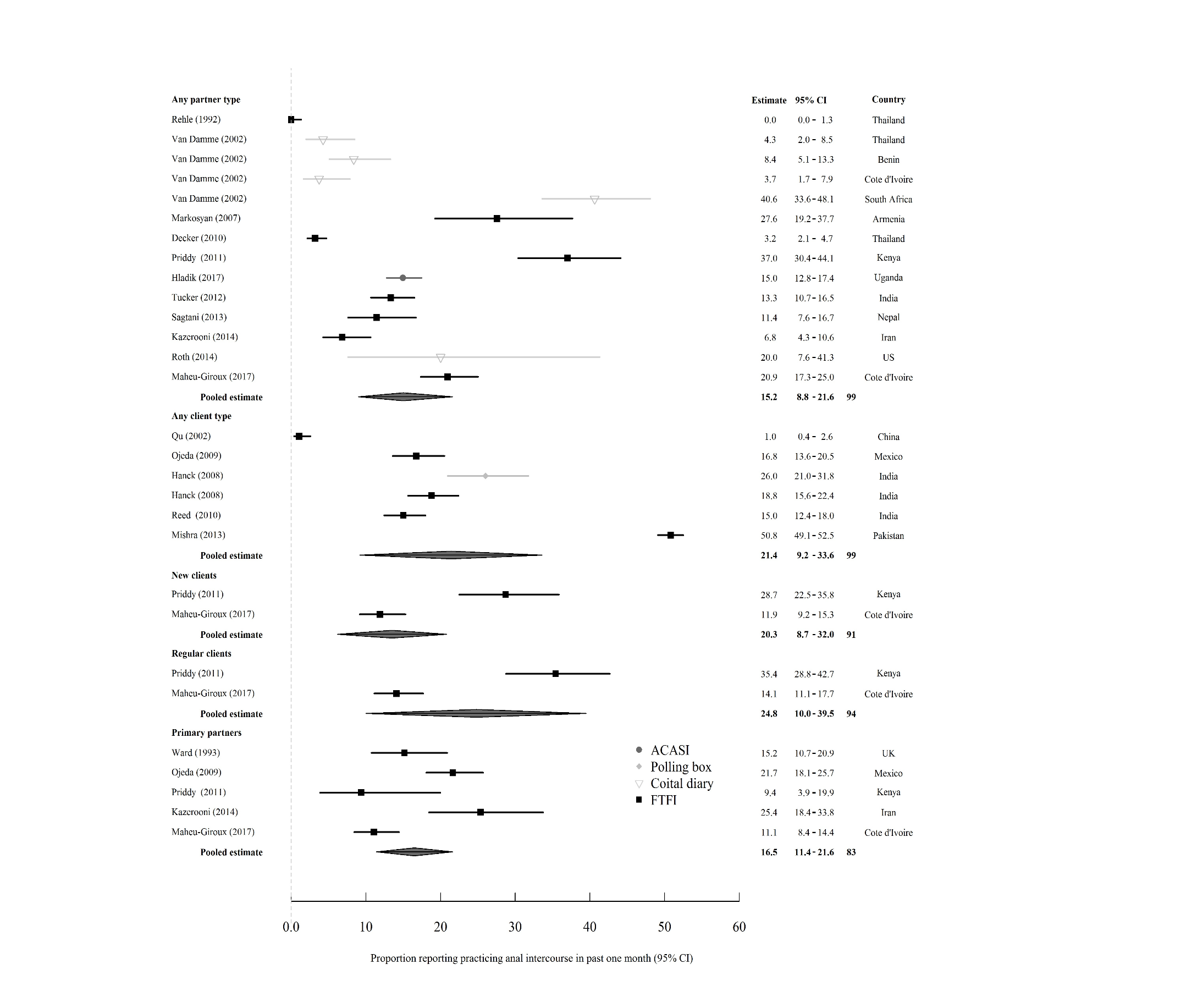 |

| Fig. S3a |
| --- |
| 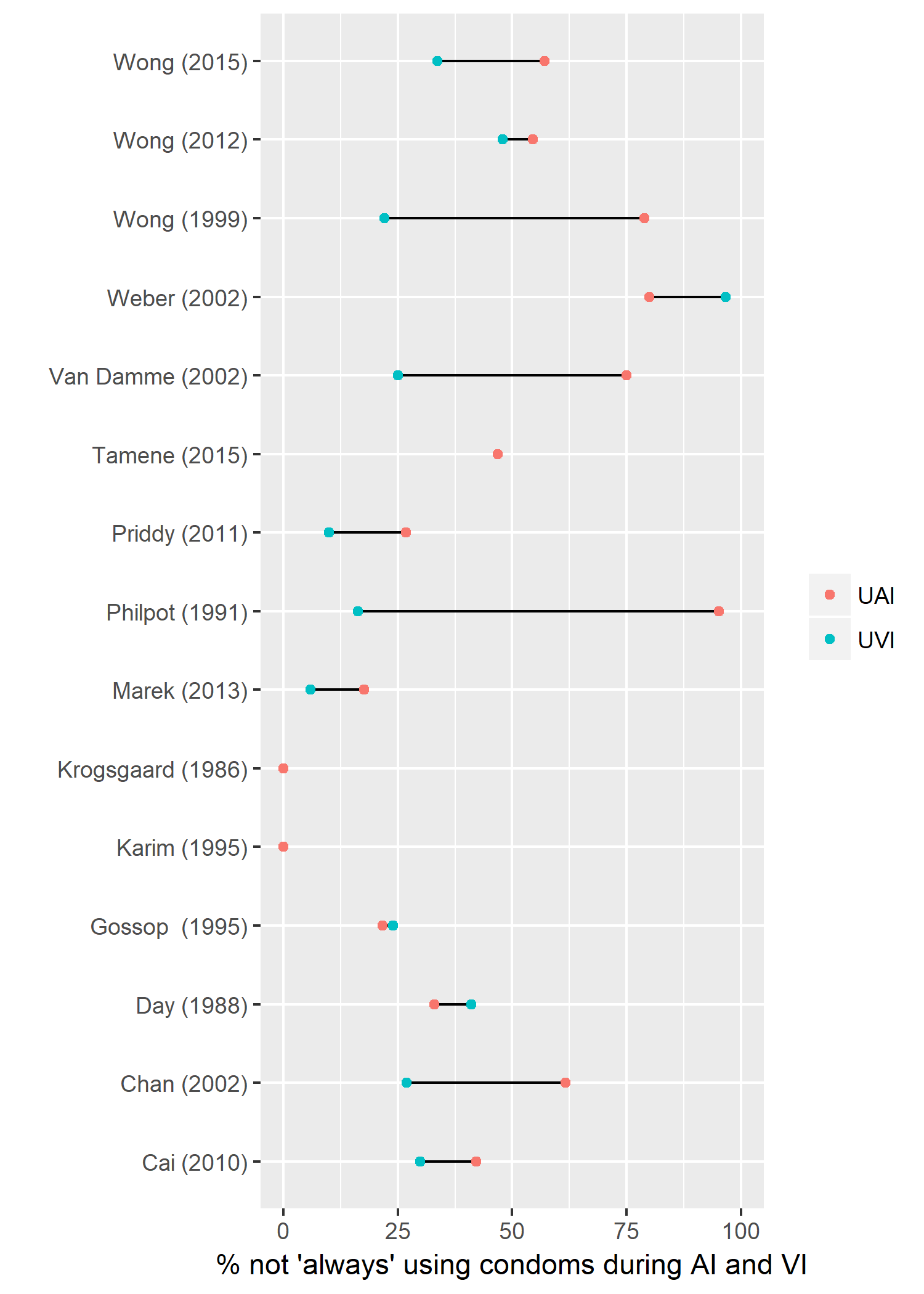 |

| Fig S3b |
| --- |
| 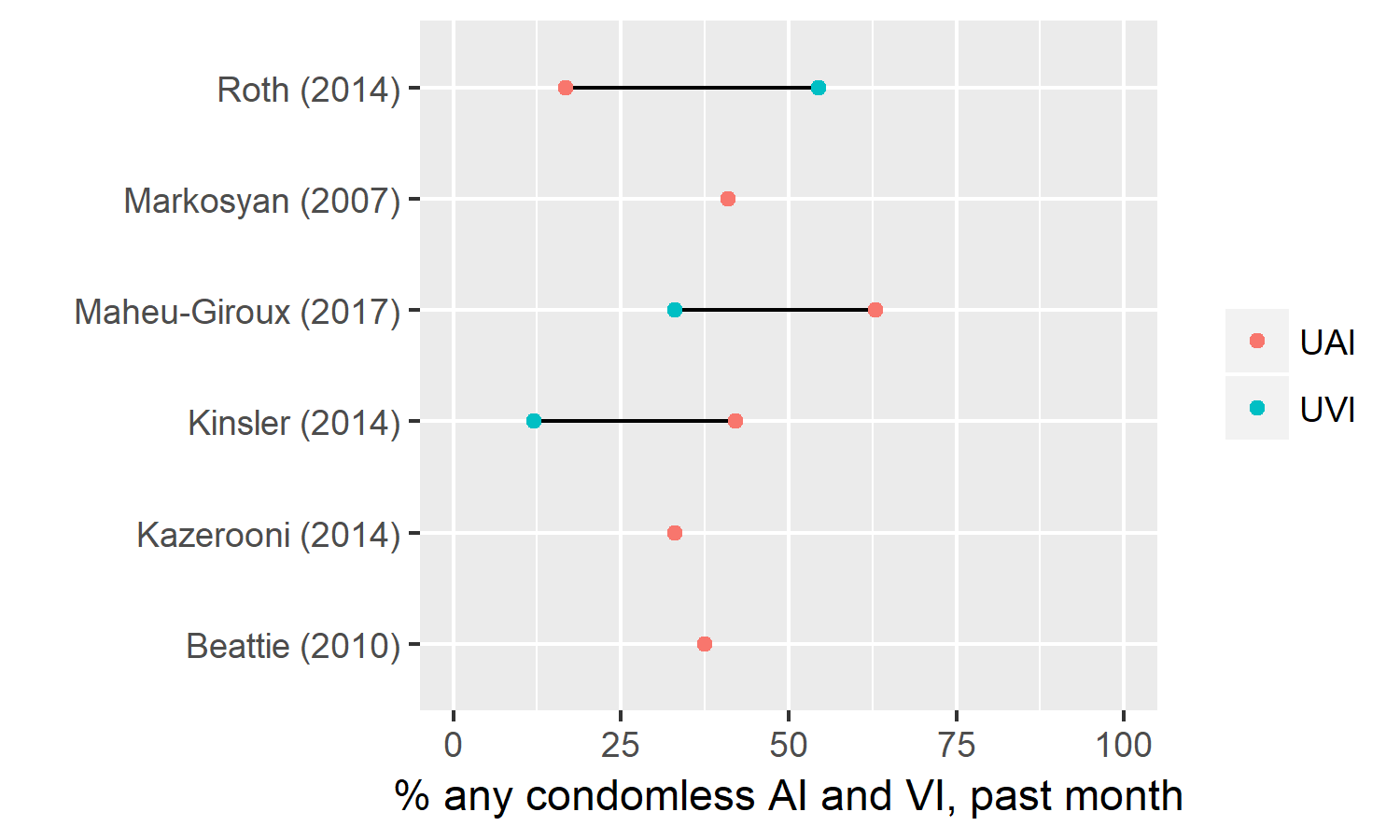 |

| Fig S3c |
| --- |
| 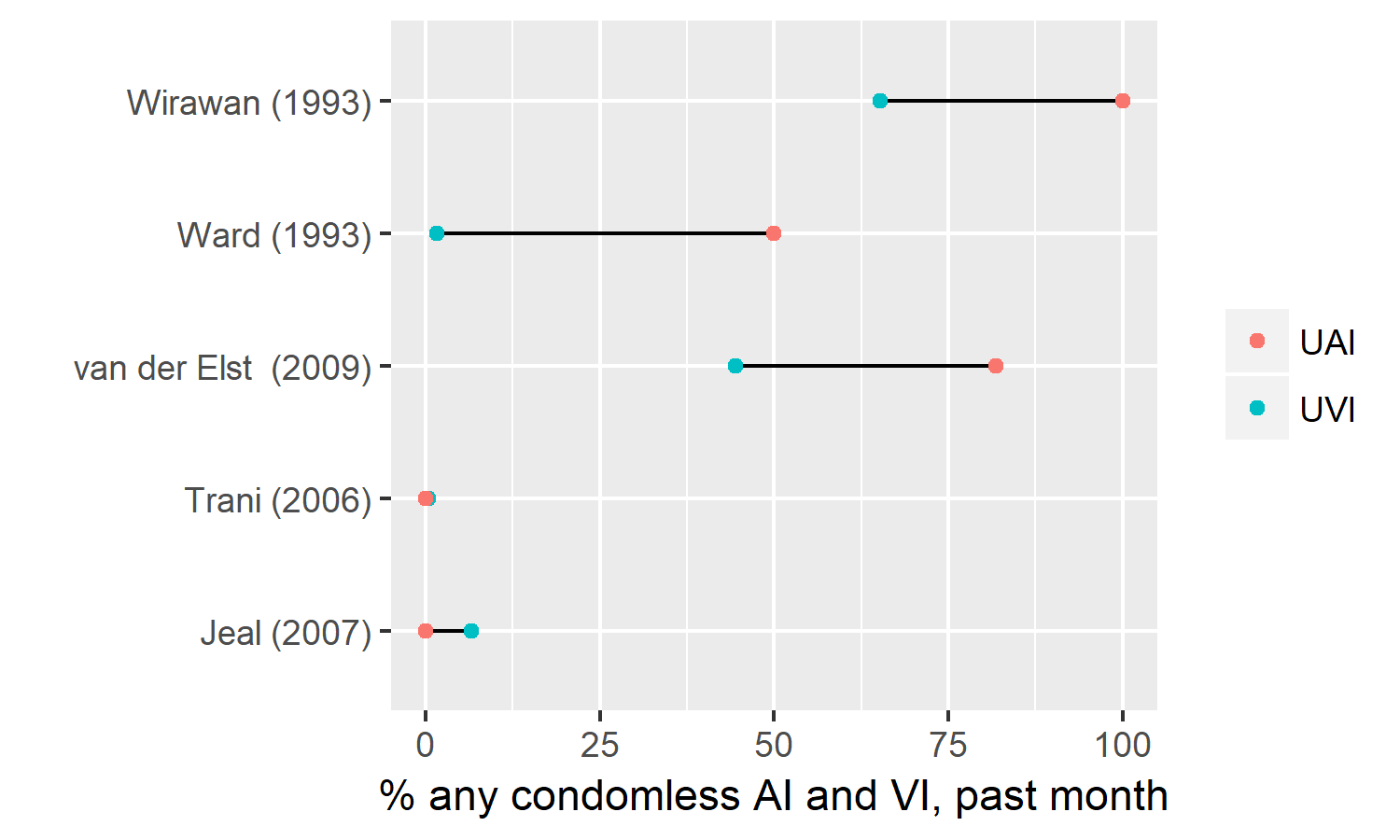 |

| Fig S3d |
| --- |
| 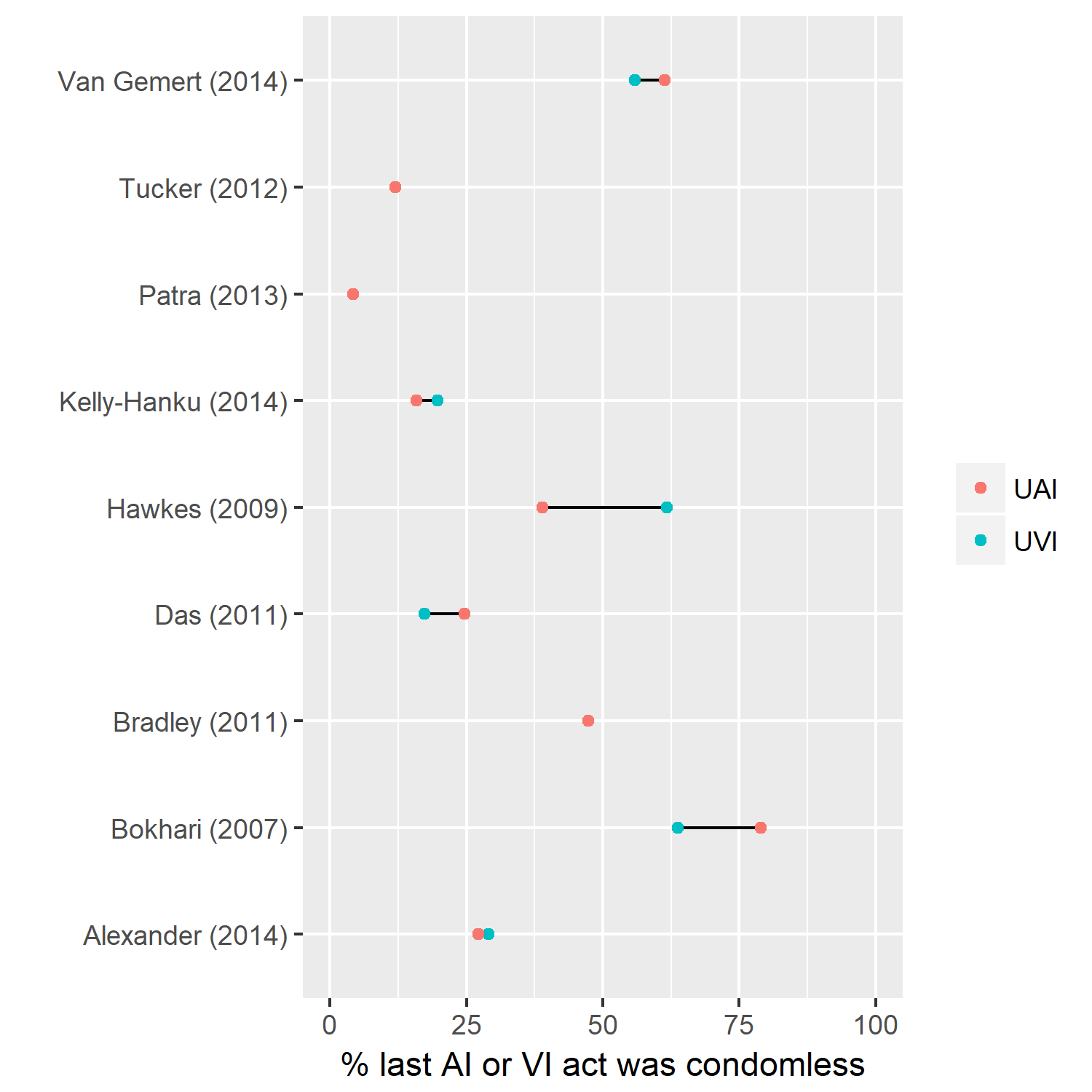 |
